# Improvement of the oxidative stability of instant fried noodles using free and microencapsulated borage (*Echium amoenum*) and black hollyhock (*Altaea rosea* var *nigra*) extracts

**DOI:** 10.1002/fsn3.3788

**Published:** 2023-10-30

**Authors:** Mahshid Zamankhani, Sohrab Moeini, Peyman Mahasti Shotorbani, Hossein MirsaeedGhazi, Afshin Jafarpour

**Affiliations:** ^1^ Department of Food Science and Technology, North Tehran Branch Islamic Azad University Tehran Iran; ^2^ Department of Food Quality Control and Hygiene, Science and Research Branch Islamic Azad University Tehran Iran; ^3^ Department of Food Technology, College of Abouraihan University of Tehran Pakdasht Tehran Iran; ^4^ Department of Food Science & Technology, Garmsar Branch Islamic Azad University Garmsar Iran

**Keywords:** antioxidants, black hollyhock, borage, encapsulation, fried noodle, oxidation

## Abstract

This study investigated the oxidative stability of instant fried noodles by applying free and microencapsulated black hollyhock extracts (BHE) and borage extracts (BE) (BE, BHE, ME‐BE and ME‐BHE). At first, the BE and BHE were encapsulated with whey protein and maltodextrin at a 90:10 ratio through a spray dryer. After evaluating particle characteristics (including anthocyanin content, zeta potential, polydispersity index (PDI), particle size, and morphology), they were added to the noodle formulation (wheat flour 78.5%, NaCl 0.78%, and water 21.21%) at 1% w/w level, and the physicochemical (proximate analysis, pH, color, cooking loss, and texture), sensory properties (taste, odor, color, texture, and overall acceptability), and oxidative stability (acid value, peroxide value, anisidine index, thiobarbituric acid index, conjugated dienes) of the fried noodles were studied. The results showed that the microcapsules had uneven shapes with angular surfaces. There was no significant difference between the zeta potential, particle size, PDI, and encapsulation efficiency of BE‐ and BHE‐loaded microcapsules, and the values reported fell between −34.96 and −34.84 mV, 1.128 and 1.195 μm, 0.247 and 0.283, and 80.08% and 83.47%, respectively. Adding extracts to the functional noodles decreased cooking loss and pH compared to the control. The noodles exhibited a darker color. BE and BHE reduced the oxidation of fried noodle oil, with microencapsulated extracts showing stronger effects during storage (*p* < .05). Sensory evaluation indicated high acceptability for all samples. Encapsulation effectively preserves the natural antioxidant activities of BE and BHE, providing potential benefits for food processing and storage.

## Graphical abstract

The oxidative stability of instant fried noodles was enhanced by using free and microencapsulated borage (BE) and black hollyhock extracts (BHE). At first, the BE and BHE were encapsulated, and the free and microencapsulated extracts were added to the formulation. The results showed that there was no significant difference between the zeta potential, particle size, polydispersity index, and encapsulation efficiency of BE and BHE microcapsules. Adding BE and BHE reduced the oxidation rate of fried noodles oil. The findings indicated that the BE and BHE can be employed as natural antioxidants to develop the oxidative stability of the instant fried noodles.

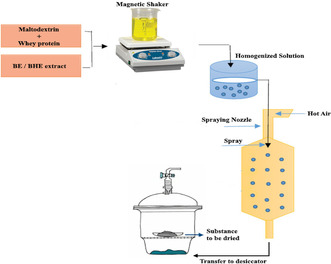

## 1 INTRODUCTION

Instant noodle is a processed and pre‐cooked food product that is popular among people from different countries because it is easy to prepare, inexpensive, and easy to store (Noonim et al., 2022). Steaming, drying, and frying processes are the main processes used to prepare instant noodles (Mu et al., 2022). Instant noodle is a popular product; however, because of the frying process for its manufacture, it might be harmful to health. The reason for this is the frying process and destructive chemical reactions such as lipid oxidation, which decrease the nutritional value and organoleptic characteristics of the product. The frying process produces reactive oxygen species (ROS) and secondary products of oxidation, which have an adverse effect on the consumer's health (Lim et al., 2017; Obadi et al., 2022). One of the most effective ways to prevent and delay lipid oxidation in food products is to use antioxidants. Due to the adverse effects of synthetic antioxidants on health, a great deal of attention has been directed toward natural antioxidants obtained from plant sources as a replacement for synthetic antioxidants (Manessis et al., 2020). Research has shown that plant extracts rich in polyphenols are potential antioxidants and can be used in food products to delay the oxidation of fats and oils (Gutiérrez‐del‐Río et al., 2021).

Borage (*Echium amoenum*), from the Boraginaceae family, is considered a valuable medicinal plant in traditional medicine, especially in Iran. The flowers are rich in polyphenols and anthocyanins (Mehran et al., 2020). This plant is used in traditional medicine for pain alleviation, pulmonary and cardiovascular diseases, influenza and infectious diseases, and cancer, as well as an antidepressant, anti‐inflammatory, and antifebrile (Zannou et al., 2021). Borage also demonstrates various functional activities such as antioxidants (Karimi et al., 2018), antivirals (Abolhassani, 2010), and antimicrobial activity (Patocka & Navratilova, 2019). The functional activities of Borage are because of the remarkable amounts of phenolic compounds, flavonoids, anthocyanins, and fatty acids (Karimi et al., 2018; Zannou et al., 2021). *Althaea rosea* or hollyhock is another valuable medicinal herb that belongs to the Malvaceae family and is famous for the special color of its flowers (Hanif et al., 2020). Research has shown that black hollyhocks are rich sources of polyphenols, anthocyanins, flavonols, carotenoids, and phenolic acids. The flowers of this herb have strong enzyme inhibitory and anti‐aging activity (Nowicka & Wojdyło, 2019). The good antioxidant and antimicrobial activity of this herb has also been reported in studies (Fahamiya et al., 2016; Hanif et al., 2020; Yourdkhani & Jafarpour, 2021).

Because of several unsaturated bonds in plant bioactive substance structures, many of the bioactive compounds are unstable and rapidly degraded when exposed to heat, light, and oxygen. One way to enhance the stability of these compounds is encapsulation, which also enables us to control their release in the body (Bai et al., 2021). Several encapsulation techniques exist, but spray drying is one of the most frequently employed methods (Ghorbanzade et al., 2017; Kaushik et al., 2015). Various combinations of proteins (gelatine, casein or caseinate, whey protein, soy protein, wheat protein, corn protein, egg white powder, etc.), carbohydrates, gums, and their derivatives (maltodextrin, highly branched cyclic dextrin, tapioca starch, waxy maize, methylcellulose, derivatized starch/glucose syrup, chitosan, gum arabic, beta‐cyclodextrin, etc.) are commonly employed as wall materials in encapsulation processes. These wall materials are often blended with other compounds such as lactose, trehalose, or lecithin (Kaushik et al., 2015). Throughout encapsulation, the sensitive and active compounds are trapped and covered using carriers of diverse materials, which also enables controlled release (Qiu et al., 2023). The use of these coating materials can enhance the stability of these compounds under environmental stressors like radiation, moisture, light, oxygen, and adverse pH conditions, as well as against the digestion process in the body (Timilsena et al., 2020; Wardhani et al., 2021). There are several applications assumable for this technology, both in the food and pharmaceutical industries (Tavakoli et al., 2021). The encapsulation process is categorized into two in terms of the scale of particles produced, including nano‐encapsulation (particles size ranging from 10 to 1000 nm) and micro‐capsulation (particles size ranging from 1 to 100 μm). These two methods can be utilized to enhance the efficiency and performance of active compounds (Shishir et al., 2018).

To the best of our knowledge, encapsulated herbal extracts have not been studied for the development of the oxidative stability of instant noodles. Therefore, in this research, the effect of free and microencapsulated borage and black hollyhock flower extracts on the physicochemical, textural, and sensory properties and the oxidative stability of instant fried noodles were investigated.

## 2 MATERIALS AND METHODS

### 2.1 Materials

Maltodextrin with DE of 4–7 and whey protein isolates were procured from Merck Co. (Germany) and Sigma Aldrich Co. (USA), respectively. Borage flowers were collected from the mountainous areas in Mazandaran Province, which were identified by the herbarium organization. Black hollyhock was purchased from Zarband Pharmaceutical Co. (Iran). Chemicals used in this research were procured from Merck Co. To produce instant noodles, wheat flour, NaCl, and sunflower oil were procured from local marketplaces in Tehran, Iran.

### 2.2 Preparation of aqueous borage and black hollyhock extracts

The borage flower and black hollyhock were dried completely in the oven at 50°C and then powdered. The extracts were extracted using ultrasound devices so that 10 g of each of the powders was homogenized with 100 mL of water and then exposed to ultrasound waves (AMMM‐M.P. Interconsulting, Switzerland) at a power of 240 W and a temperature of 20°C for 10 min. The tubes containing solutions were covered by aluminum foil and then placed in a shaker for 24 h. Finally, the filtered extracts were kept at 4°C until further use (Rabiei et al., 2012).

### 2.3 Total phenol, total flavonoid, and antioxidant activity of borage and black hollyhock extracts

The total phenolic content (TPC) in the extract was determined using the Folin–Ciocalteu method. A 0.2 μL sample solution (1 mg/mL) was added to a test tube containing 1 mL of Folin–Ciocalteu's reagent and 2 mL of Na_2_CO_3_ (7.5%). The final volume was adjusted to 7 mL with deionized water. After 2 h of incubation at room temperature, the absorbance was measured at 765 nm using a spectrophotometer (Photonix Ar 2015). The TPC was expressed as milligrams of gallic acid equivalents (GAE) per gram of extract (mg GAE/g extract) (Zengin et al., 2011). The total flavonoid content (TFC) of the extracts was determined as follows (Adel Pilerood & Prakash, 2014). A 5.0 mL solution of 2% aluminum trichloride (AlCl_3_) in methanol was mixed with an equal volume of the extract solution (10 mg/mL). Absorption readings at 415 nm were taken using a spectrophotometer after 10 min, with a blank sample consisting of the extract solution mixed with 5.0 mL of methanol without AlCl_3_. The TFC was calculated using a standard curve with quercetin and expressed as grams of quercetin equivalents per 100 g of the sample.

The antioxidant activity of borage extracts (BE) and black hollyhock extract (BHEs) was evaluated using the DPPH (2,2‐diphenyl‐1‐picrylhydrazyl) assay through two spectrophotometric methods. Different concentrations of sample extracts (0.3 mL) were mixed with a methanolic solution containing DPPH radicals (6 × 10–5 mol/L, 2.7 mL). The mixture was vigorously shaken and kept in the dark until stable absorption values were achieved. The reduction of the DPPH radical was measured by continuously monitoring the decrease in absorption at 517 nm. The DPPH scavenging effect was calculated as the percentage of DPPH discoloration using the provided equation:

$$\%\text{Scavenging effect}=\left[\left(\text{ADPPH}-\mathrm{AS}\right)/\text{ADPPH}\right]\times 100$$

where AS is the absorbance of the solution when the sample extract has been added at a particular level and ADPPH is the absorbance of the DPPH solution. BHA and tocopherol were used as reference compounds (Oliveira et al., 2008). The total antioxidant potential of the plant extracts was observed using a ferric reducing antioxidant power (FRAP) assay. A spectrophotometric method was used to measure the reducing power. Different concentrations of extracts were mixed with 2.5 mL of phosphate buffer (0.2 M, pH 6.6) and 2.5 mL of 1% potassium ferricyanide (10 mg/mL). The mixture was incubated at 50°C for 20 min, then rapidly cooled, mixed with 2.5 mL of 10% trichloroactic acid, and centrifuged at 4000 g for 10 min. The supernatant (2.5 mL) was mixed with distilled water (2.5 mL), and then ferric chloride (0.5 mL, 0.1%) was added and allowed to stand for 10 min. The absorbance was read spectrophotometrically at 700 nm (Adel Pilerood & Prakash, 2014).

### 2.4 Microencapsulation of BE and BHE

**FIGURE 1:**
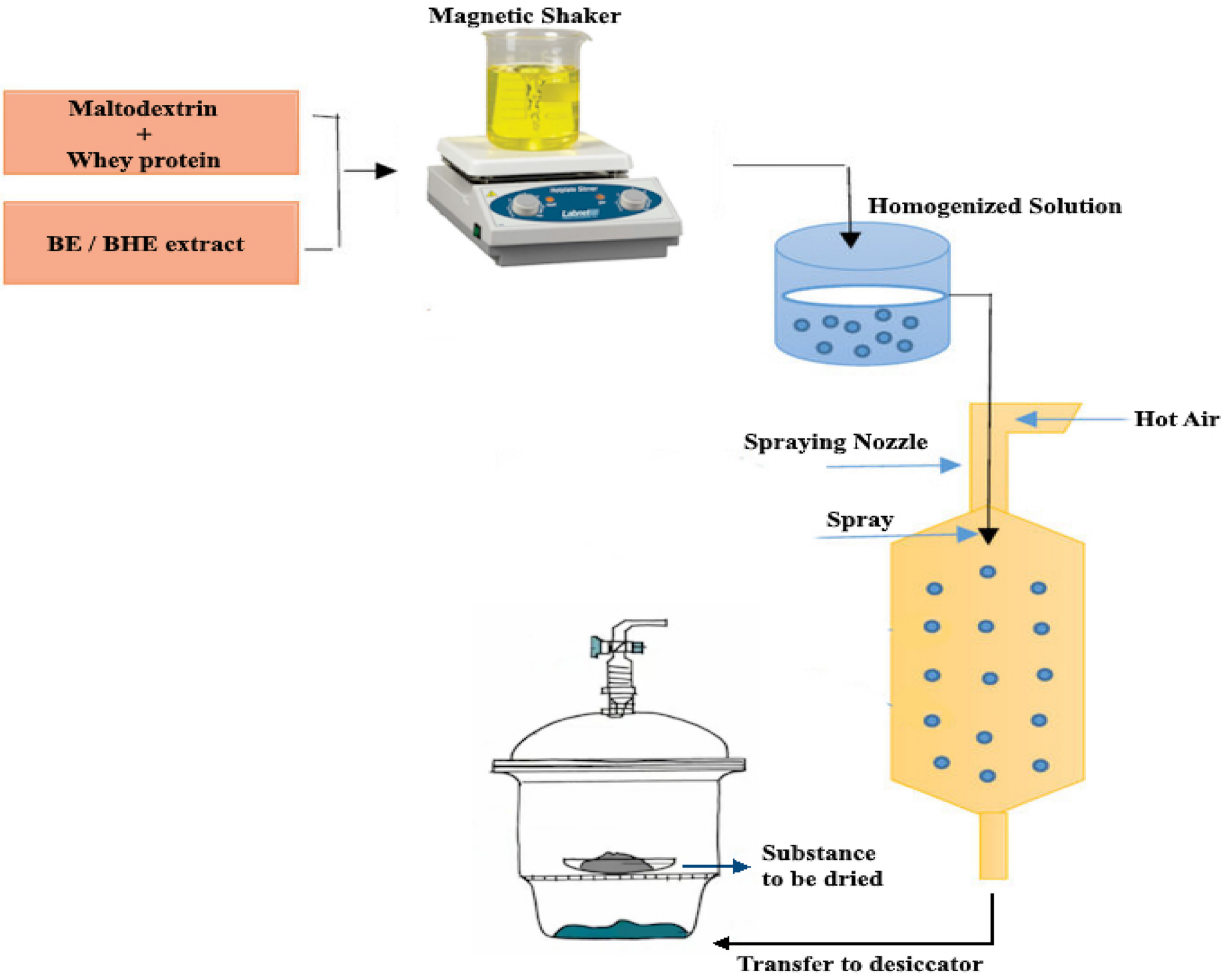
Process flow chart for the encapsulation of borage extract and black hollyhock extract in whey protein and maltodextrin wall material.

Carrier materials, including the mixture of maltodextrin and whey protein, were used at a 10:90 ratio. The carrier materials and extracts (BE or BHE) were mixed together and dissolved in distilled water containing 0.02% preservative (w/w; sodium azide) using a magnetic stirrer (95 g, 45°C, 30 min). Afterward, the solutions were homogenized for 2 min at 16060 g and for 10 min at 45995 g, and then immediately used to prepare the capsule powder. A two‐flowing nozzle dryer (Counter‐current, Iran) was used to dry the microencapsulated extracts. The solutions were injected into the spray dryer by a pump (400 kPa air pressure). The inlet and outlet air temperatures were 180 and 80°C, respectively. The produced powders were collected in the subcircumference chamber and immediately transferred to the desiccator for cooling. Finally, BE and BHE microcapsule powders were poured into glass jars covered with aluminum foil and kept at 4°C (Bae & Lee, 2008) (Figure 1).

#### 2.4.1 BE and BHE microcapsule characterization

Microparticle morphology was examined using a scanning electron microscope (SEM; LABOMED LX400, USA), after applying a 15 nm gold coating to the samples (Baltrusch et al., 2022). The mean particle size and polydispersity index (PDI) of the microcapsules were measured using dynamic light scattering using a Zetasizer (nano‐Zs, Malvern, England). Particle size was expressed as Z average hydrodynamic diameteZeta potential was also measured using Zetasizer at 25°C. The encapsulation efficiency (EE) (%) of microcapsules was measured based on the ratio of the polyphenols retained in the microcapsules to the amount of polyphenols in the extracts at the beginning of the encapsulation process (Qiu et al., 2023). The anthocyanin content of microcapsules was determined using the ammonia‐HCl method (Egbuna et al., 2018). Anthocyanins were extracted using ethanolic HCl and measured at the wavelength of peak absorption. A 2.0 g sample was mixed with 70 mL of ethanolic HCl and stored overnight at 4°C. The extract was filtered and made up to 100 mL. For spectrophotometric measurement, 1.0 mL of the sample was diluted to 10 mL. The optical density (OD) was determined after 2 h of dark storage, and the color was measured at the peak absorbance of 545 nm.

### 2.5 Instant fried noodles

The control noodle formulation consisted of wheat flour (400 g), NaCl (4 g), and distilled water (108 mL). In the enriched noodles formulation, 1% of wheat flour was substituted for the powder of free and microencapsulated BE and BHE. The ingredients of the formulation were mixed using a mixer for 7 min to obtain a uniform paste. After passing through two rotating parallel rollers, the dough was turned into a dough belt, and then this dough passed through the rollers and turned into a thin sheet, which was cut immediately by a stringer. Then the dough strands were passed through a steam tunnel on a conveyor for 5 min at 90°C to gelatinize the starch and improve the texture of the noodles. The noodles were then placed in a mold and fried for 3 min in a hot oil tank at a temperature of 140°C. Finally, the noodles were cooled down to the ambient temperature, packed in 100 g packages, and stored in a dry, cold place away from direct sunlight and at room temperature (23 ± 2°C) for 120 days (Khalkhali & Mostaghim, 2022).

### 2.6 Physicochemical properties of noodles

Protein, moisture, fat, and ash contents of the noodles were measured using AACC approved methods 44‐15, 46‐10, 30‐26, and 08‐03, respectively (American Association of Cereal Chemists, 2000). The carbohydrate content was measured following AOAC 948.02 (AOAC, 1990). The pH of the noodles was measured using a digital pH‐meter. The cooking loss of the noodles was determined according to the method described by Özyurt et al. (2015). A 10 g noodle sample was boiled in 300 mL of distilled water. After 10, 20, and 30 min, the samples were taken out, rinsed with distilled water, and drained for 2 min. The cooking loss of the noodle samples was calculated using the following equation:

$$\text{Cooking loss}\;\left(\%\right)=\frac{\text{Weight of drained residue in cooking water}}{\text{Weight of uncooked noodle}}\times 100$$

In addition, the color parameters of noodles, including redness‐greenness (a*), lightness (L*), yellowness‐blueness (b*), and total color difference (Δ*E*) were determined using the Hunterlab (Color flex, USA) (Xu et al., 2019). After production, three small dough pieces (8 cm × 8 cm) were cut from the final dough sheet and placed on a whiteboard. Each dough piece was measured twice on different sides at 0 and 24 h. Color measurements were averaged from eight individual determinations. The dried noodles were ground using an Alpha Tech grinder and passed through a 28‐mesh sieve. The color of the resulting noodle powder was measured using a granular material attachment.

### 2.7 Texture profile analysis (TPA) of noodles

The texture profile of the noodles was studied using a Texture Analyzer (CT310k Texture, Brookfield, USA). A round plate probe (3.5 cm diameter) with a speed of 60 mm/min and a cell of 5 N was used. The samples were compressed to 30% of their initial height at room temperature. The textural parameters measured were hardness (N), adhesiveness, springiness (cm), gumminess (N), and chewiness (N/cm) (Han et al., 2011).

### 2.8 Oxidation indexes of noodles

The noodle oil was extracted using the Soxhlet method at 40°C for 6–8 h using petroleum ether as solvent and finally the solvent, was evaporated by a rotary evaporator (Heidolph, Germany). Acid value (AV) was determined following Mazaheri et al. (2019) and reported as mg KOH per g of oil. Peroxide value (POV) was measured using titration methods and reported as meq O_2_ per kg of oil (Drinić et al., 2020). The thiobarbituric acid (TBA) index was determined using the spectrophotometer method at 532 nm wavelength and reported as mg of malondialdehyde per kg of oil (mg MDA/kg) (Afshari & Sayyed‐Alangi, 2017). Anisidine index (AI) was determined using the spectrophotometer method at 350 nm wavelength (Chen et al., 2014). Conjugated dienes (CDs) were measured using a spectrophotometer method at 234 nm and reported as mmol/kg (Delfanian et al., 2015).

### 2.9 Sensory evaluation of noodles

Sensory evaluation of noodles was carried out through the 5‐point Hedonic method (5: very good, 1: very bad) and by administering the questionnaire to 30 untrained panelists. The sensory characteristics investigated in this research included taste, odor, color, texture, and overall acceptability.

### 2.10 Statistical analysis of data

Data analyses were performed three times for all the samples and experiments, and the results were reported as mean ± SD. The Duncan multi‐range post hoc test and one‐ way ANOVA analysis were used to analyze the data at a significance level of *p* < .05. Data analysis was done using SPSS Statistics 22.0 (Chicago, USA).

## 3 RESULTS AND DISCUSSION

### 3.1 Total phenol and flavonoid content and antioxidant activity of BE and BHE

Phenolic and flavonoid compounds are the major secondary metabolites in the plants produced in response to environmental stress, and some of these compounds have a high ability to neutralize free radicals due to their hydroxyl groups and good antioxidant activity (Fukumoto & Mazza, 2000). The TPC and TFC of BE and BHE obtained in this study are presented in Table 1. The TPC of BE and BHE was 19.57 and 23.07 mg GAE/g DW, respectively, and the TFC was 18.48 and 16.73 mg QE/g DW, respectively. The TPC of BHE was higher than that of BE, while BE had a higher TFC (*p* < .05). These values were generally higher than the values reported by Karimi et al. (2018) and Nowicka and Wojdyło (2019). Karimi et al. (2018) found gallic acid, caffeic acid, salicylic acid, pyrogallol, rutin, daidzein, and myricetin as the major bioactive compounds of BE. In another study, the presence of various bioactive compounds, including polyphenols, carotenoids, flavonoids, vitamin C, and anthocyanins in borage flowers was confirmed (Adel Pilerood & Prakash, 2014). Ferulic acid, vanillic acid, syringic acid, *p*‐coumaric acid, *p*‐hydroxybenzoic acid, *p*‐hydroxyphenylacetic acid, and caffeic acid were found in BHE (Dudek et al., 2006). Ahmad et al. (2016) reported the presence of flavonoids, teriterpenoids, alkaloids, saponins, tannins, steroids, and phenolic acid in the BHE.

**TABLE 1:** Comparison of the total flavonoid (TFC), total phenol (TPC), total anthocyanin (TAC), and antioxidant activity of the BE and BHE.

Samples	TPC (mg GAE/g DW)	TFC (mg QE/g DW)	IC50 (μg/mL)	FRAP (absorbance at 700 nm)
BE	19.57 ± 0.66^b^	18.48 ± 0.21^a^	136.08 ± 3.08^b^	1.61 ± 0.06^b^
BHE	23.07 ± 0.98^a^	16.73 ± 0.59^b^	150.80 ± 1.19^a^	2.19 ± 0.08^a^

*Note*: Values represent the mean (*n* = 3) ± SD. Different letters in each column show a significant difference (*p* < .05).

Abbreviations: BE, borage extract; BHE, black hollyhock extract; FRAP, ferric reducing antioxidant power.

Antioxidant activity is a major function of herbal extracts and essential oils because oxidation can have a very destructive effect on biological substances and many diseases such as diabetes, digestive disorders, cancer, Alzheimer's, Parkinson's, etc. (Rezaei Savadkouhi et al., 2020). There are many methods to determine the antioxidant activity of plant extracts. In this research, the antioxidant activity of BE and BHE was measured by DPPH radical scavenging and FRAP methods (Table 1). One of the most widely used methods to study the antioxidant activity of herbal extracts, essential oils, and their bioactive compounds is the DPPH free radical scavenging method, which is inexpensive and simple (Diniz do Nascimento et al., 2020). The FRAP is another method to determine the antioxidant activity of antioxidants, which demonstrates the ability of bioactive agents to reduce ferric iron (Fe^3+^) to ferrous iron (Fe^2+^) (Esmaeili et al., 2021). In general, the higher the reducing power, the higher the tendency to donate electrons (Ganji & Sayyed‐Alangi, 2017). The IC50 values of BHE (150.80 μg/mL) were significantly higher than those of BE (136.08 μg/mL), so BE had a higher antiradical activity than BHE. However, the reduced power of BHE (2.19 absorbance at 700 nm) was significantly higher than BE (1.61 absorbance at 700 nm). The types of active compounds in herbal extracts and their structures have a remarkable effect on their antioxidant activity. Therefore, due to the difference in the type and ratio of active compounds in the BE and BHE, as well as the structural differences of these active compounds, a significant difference was observed in the antioxidant activity of these two extracts. Karimi et al. (2018) reported the DPPH radical scavenging and the FRAP of the water extract of borage flower equal to 290.3 μg/mL and 282.2, respectively. In another study, the DPPH radical scavenging and FRAP of BE were reported to be equal to 73.58% and 477.4 μmol/g, respectively (Borowy et al., 2017). Generally, the difference in the content of bioactive compounds and the antioxidant activity of the extracts reported by studies is related to the difference in the environmental and weather conditions, the harvesting time, as and the measurement method (Asadi‐Samani et al., 2014; Zemmouri et al., 2019).

### 3.2 The characteristics of BE‐ and BHE‐loaded microcapsules

**FIGURE 2:**
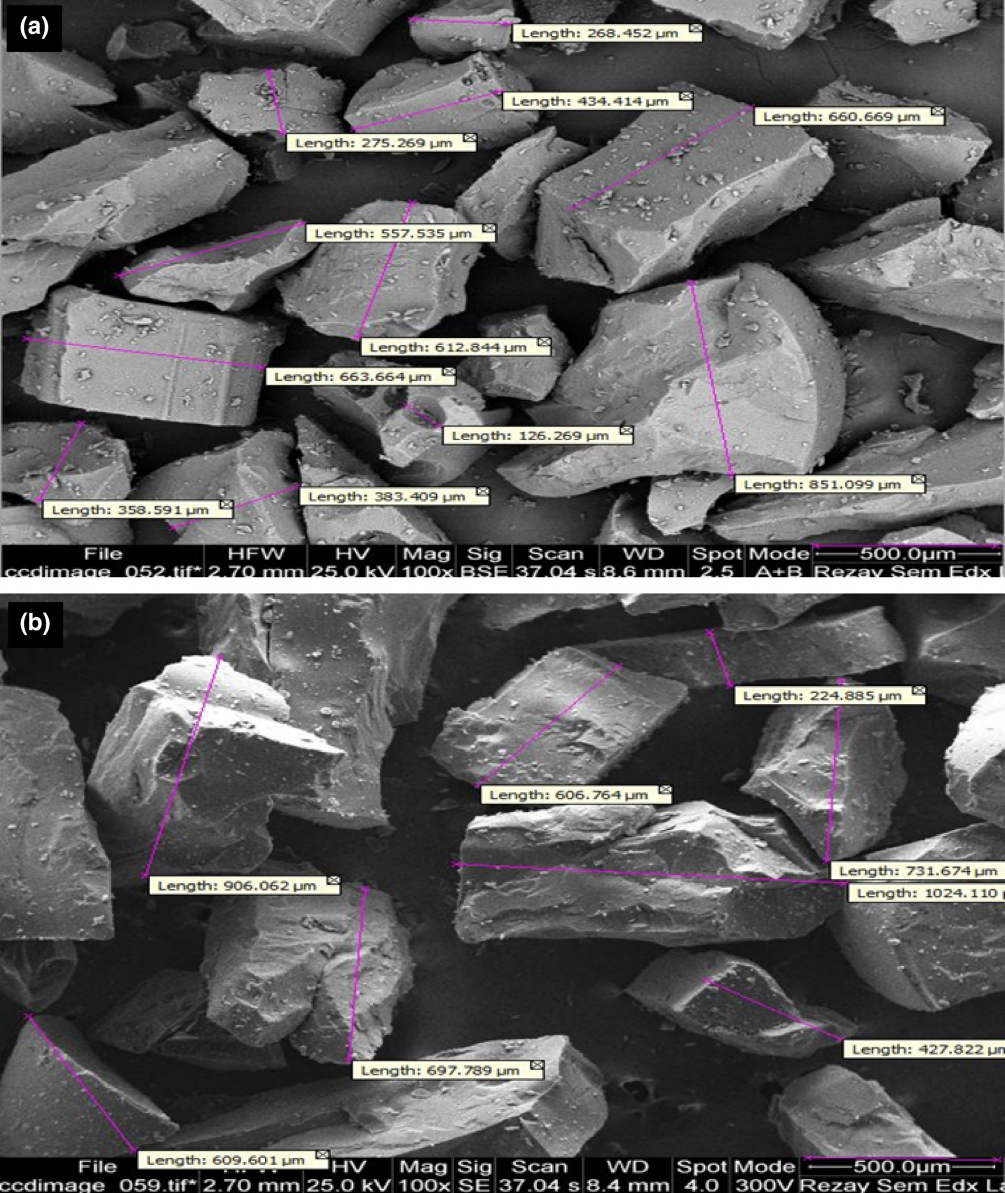
Scanning electron microscopic images of (a) microencapsulated borage extract (ME‐BE), and (b) microencapsulated black hollyhock extract (ME‐BHE).

The SEM images of the BE‐ and BHE‐loaded microcapsules are shown in Figure 2. The microcapsules obtained did not exhibit a distinct geometric shape, likely due to the spray drying mechanism. Generally, the size of microcapsules can be adjusted by altering various spray drying conditions, including the direction and temperature of the inlet air, initial and final moisture levels of the particles, type and concentration of the coating material, and viscosity of the feed (Akbarbaglu et al., 2019). Furthermore, the irregular shape and varying particle sizes could be attributed to the aggregation and adhesion of smaller particles caused by the presence of maltodextrin, aligning with findings from previous studies (Akbarbaglu et al., 2019; Hu et al., 2016). Microcapsules were prepared in optimum conditions with maltodextrin and whey protein in cubic form, some of which have trituration on the surface. The wrinkle at the surface of the microcapsules can be attributed to the high temperature of the drying inlet and the rapid surface water evaporation (Drusch & Berg, 2008). These wrinkles are part of the characteristics of the microcapsules prepared by the spray drying method (Ré, 1998). According to other studies, the formation of wrinkles is due to the rapid formation of the shell and the swelling in the particles due to increased particle temperature and vapor pressure (Nijdam & Langrish, 2006). Despite the high temperatures employed in the spray drying method, the use of shorter operating times minimizes damage to the active compounds, making it a suitable technique for heat‐sensitive bioactive compounds (Navarro‐Flores et al., 2020).

The physicochemical characteristics of BE and BHE microcapsules are listed in Table 2. Particle size is an important factor that plays a noticeable role in particle stability, so that by reducing particle size, the stability of colloidal systems increases (Homayoonfal et al., 2021). The mean particle sizes of BE and BHE microcapsules were 1.195 and 1.128 μm, respectively, and there was no significant difference in the particle sizes of these two microcapsules. PDI demonstrates the homogeneity of particles, and the lower values (especially less than 0.3) are more favorable and indicate higher homogeneity (Piran et al., 2020). The PDI values of BE and BHE microcapsules were 0.247 and 0.283, respectively, and there was no significant difference between the two samples. Zeta potential expresses the surface charge of particles and also shows the stability of particles (Homayoonfal et al., 2021). In terms of zeta potential, no significant difference was observed between the two microcapsule samples, and the average values of BE and BHE microcapsules were −34.84 and −31.96 mV, respectively. Probably, the use of the same coating materials to prepare these capsules is the reason for the lack of significant differences between them. In general, particles with zeta potentials higher than 30 mV have remarkable stability and indicate a low tendency to accumulate (Lu et al., 2014). Therefore, the microcapsules produced in this research had good stability. Carboxylate groups are present in whey protein isolate, which is the reason for the negative charge of the particles prepared with it (Soleimanifar et al., 2020). Similarly, Yazdan‐Bakhsh et al. (2021) reported that the encapsulated *Heracleum lasiopetalum* extract with whey protein isolate had a negative charge and a high zeta potential value (−41.72 mV). The zeta potential of bene hull extract capsules prepared with whey protein and gum was also negative (−29.20 mV) (Delfanian et al., 2018). The microcapsules produced in this research also had a high EE and the values of EE of the BE and BHE microcapsules were 80.08% and 83.47%, respectively. Since whey protein isolate has high emulsifying properties and has both hydrophilic and hydrophobic parts in its structure, it has a high EE (Hosseinnia et al., 2017). An EE of more than 70% was observed for the encapsulation of date palm pit extract and saffron extract with whey protein isolate (Esfanjani et al., 2015).

**TABLE 2:** The mean particle size, PDI, zeta potential, EE, and anthocyanin content of BE‐ and BHE‐loaded microcapsules.

Samples	Mean particle size (μm)	PDI	Zeta potential (mV)	EE (%)	Anthocyanin content (mg/g)
BE microcapsules	1.195 ± 0.046^a^	0.247 ± 0.033^a^	−34.84 ± 2.03^a^	80.08 ± 3.29^a^	115.70 ± 6.86^a^
BHE microcapsules	1.128 ± 0.029^a^	0.283 ± 0.018^a^	−31.96 ± 1.81^a^	83.47 ± 1.54^a^	71.91 ± 5.91^b^

*Note*: Values represent the mean (*n* = 3) ± SD. Different letters in each column show a significant difference (*p* < .05).

Abbreviations: BE, borage extract; BHE, black hollyhock extract; EE, encapsulation efficiency; PDI, polydispersity index.

The anthocyanin content of BE and BHE microcapsules was also determined in this research, and its results are given in Table 2. The anthocyanin content of BE microcapsule (115.70 mg/g) was significantly higher than that of BHE microcapsule (71.91 mg/g) (*p* < 0.05). Generally, various bioactive compounds are present in plant extracts, including BE and BHE, at different quantities. The anthocyanin content of borage in Borowy et al. (2017) study was equal to 0.68 mg/100 g. However, Adel Pilerood and Prakash (2014) showed that the anthocyanin content of this flower was equal to 104.4 mg/100 g. Hosaka et al. (2012) reported the presence of high amounts of anthocyanins in black hollyhock. In another study, the anthocyanin content of black hollyhock was found to be 286.14 mg/100 g (Nowicka & Wojdyło, 2019).

### 3.3 Physicochemical properties of instant fried noodles

The physicochemical properties of fried noodles are presented in Table 3. The moisture, protein, fat, ash, and carbohydrate content of the samples were in the 8.99%–9.73%, 11.86%–12.47%, 9.87%–10.49%, 1.00%–1.44%, and 69.51%–69.91% ranges, respectively. In terms of moisture, fat, and carbohydrate content, no significant statistical difference was found between the noodle samples. Adding free BE and BHE also had no significant effect on the protein and ash content of the noodles, while adding the microencapsulated form resulted in a significant increase in the protein and ash content of the samples (*p* < .05). This increase is probably related to the use of whey protein isolate and maltodextrin coatings. The pH of the fried noodle samples was in the 7.33–7.58 range, and adding free and encapsulated BE and BHE resulted in a significant decline in the pH of the noodles. This reduction is related to the presence of acidic compounds, such as phenolic acids, in these extracts. Adding *Cosmos caudatus* Kunth. (*Ulam Raja*) aqueous and dry extract to the noodle formulation also significantly decreased the pH of the samples compared to the control group (Norlaili et al., 2014). A decrease in the pH of cooked noodles due to the increase in the level of pomegranate peel extract was also reported by Kazemi et al. (2017), which was attributed to the acidic pH of this extract.

**TABLE 3:** Physicochemical properties of instant fried noodle samples.

Samples	Moisture (%)	Protein (%)	Fat (%)	Ash (%)	Carbohydrate (%)	pH
Control	9.06 ± 0.26^a^	12.03 ± 0.20^b^	10.49 ± 0.35^a^	1.04 ± 0.07^b^	69.51 ± 0.26^a^	7.58 ± 0.02^a^
BE	9.10 ± 0.18^a^	11.86 ± 0.29^b^	10.23 ± 0.62^a^	1.09 ± 0.06^b^	69.64 ± 0.18^a^	7.39 ± 0.04^b^
BHE	8.99 ± 0.30^a^	12.09 ± 0.33^b^	9.87 ± 0.40^a^	1.00 ± 0.04^b^	69.55 ± 0.24^a^	7.34 ± 0.02^b^
ME‐BE	9.65 ± 0.37^a^	12.47 ± 0.14^a^	9.91 ± 0.52^a^	1.37 ± 0.08^a^	69.73 ± 0.42^a^	7.33 ± 0.02^b^
ME‐BHE	9.73 ± 0.45^a^	12.45 ± 0.17^a^	9.87 ± 0.49^a^	1.44 ± 0.06^a^	69.91 ± 0.39^a^	7.36 ± 0.01^b^

*Note*: Values represent the mean (*n* = 3) ± SD. Different letters in each column show a significant difference (*p* < .05).

Abbreviations: BE, borage extract; BHE, black hollyhock extract; ME‐BE, microencapsulated borage extract; ME‐BHE, microencapsulated black hollyhock extract.

### 3.4 Cooking loss of instant fried noodles

**FIGURE 3:**
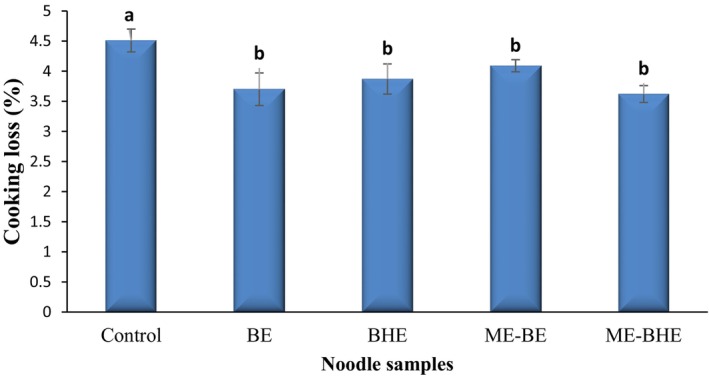
Cooking loss values (%) of instant fried noodle samples. Bars represent the mean (*n* = 3) ± SD. Different letters on the bars show a significant difference (*p* < .05). BE, borage extract; BHE, black hollyhock extract; ME‐BE, microencapsulated borage extract; ME‐BHE, microencapsulated black hollyhock extract.

Cooking loss is an important quality parameter in noodles and indicates the leakage of solids from the product during the cooking process (Kang et al., 2017). The results of the cooking loss of fried noodles are presented in Figure 3. Adding free and microencapsulated BE and BHE resulted in a significant decrease in the cooking loss of the noodle samples (*p* < .05), so that the cooking loss of the control sample was 4.51% versus 3.62%–4.09% in the samples enriched with the extracts. No statistically significant difference was found between the cooking loss values of noodle samples containing free and microencapsulated extracts. Generally, a good‐quality noodle has a cooking loss of less than 8% (Norlaili et al., 2014), and based on this, all the noodle samples produced in this research had a good quality. The decrease in the cooking loss of noodles due to free and microencapsulated extracts can be explained by the creation of complex networks between protein and polyphenolic compounds, which improved the textural properties of the noodles (although texture was improved in this research but the results were not statistically significant) and caused a reduction in cooking loss (Lee et al., 2016). Additionally, the study revealed that decreasing the pH of noodles can effectively reduce cooking loss by reducing the solubility of γ‐ and α‐gliadins (Kazemi et al., 2017). In this study, the inclusion of both free and encapsulated extracts led to a decrease in the pH of the noodles compared to the control sample. This decrease in pH may account for the observed reduction in cooking loss of the fortified noodles. These findings are in agreement with Marinelli et al. (2015). In agreement with these results, Song and Yoo (2017) reported that green tea extract and pea protein isolate decreased the cooking loss of instant fried noodles. In line with these results, a reduction in noodle cooking loss due to the incorporation of 2% and 4% levels of *Cosmos caudatus* Kunth (*Ulam Raja*) aqueous and dry extracts was reported by Norlaili et al. (2014). However, Widyawati et al. (2022) found that adding less *Pluchea indica* leaf tea did not have a significant effect on noodle cooking loss.

### 3.5 Color properties of instant fried noodles

**FIGURE 4:**
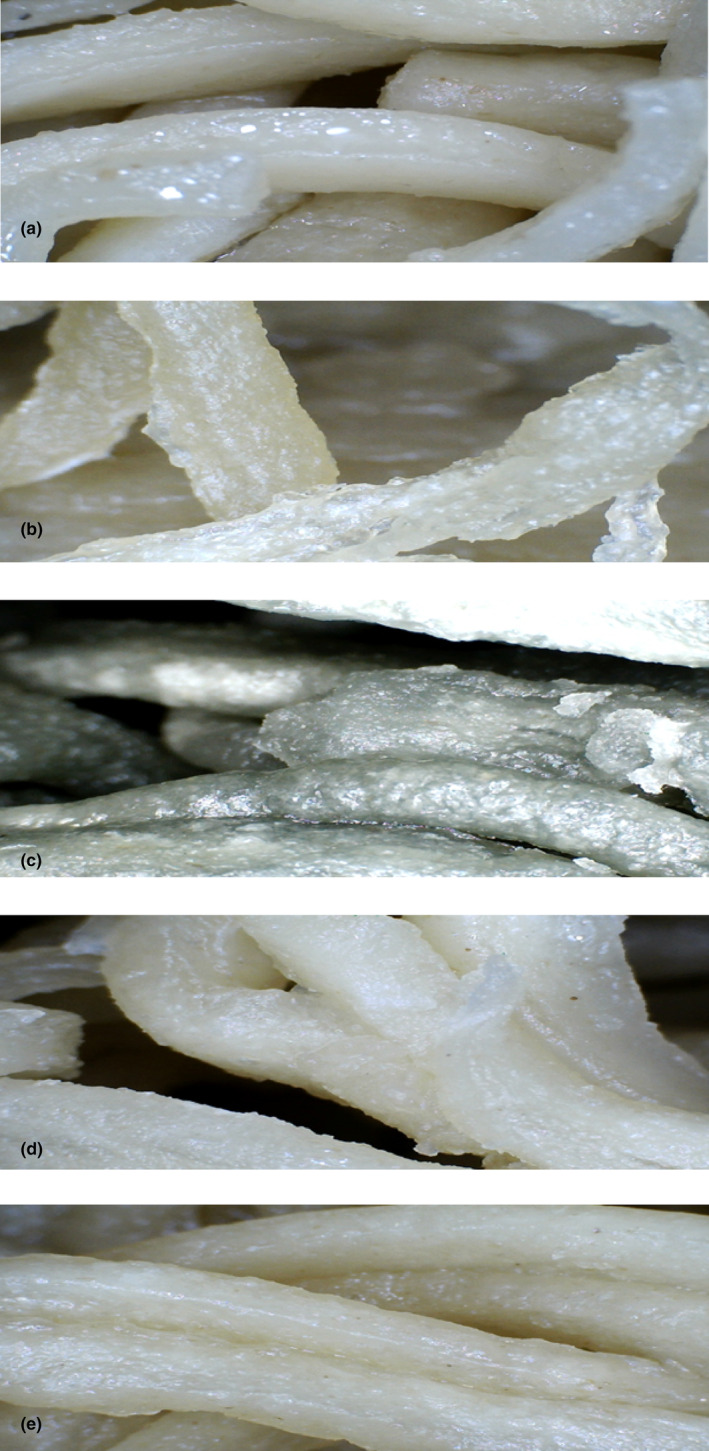
Color of instant fried noodle samples: (a) control; (b) noodle containing BE; (c) noodle containing BHE; (d) noodle containing ME‐BE; and (e) noodle containing ME‐BHE. BE, borage extract; BHE, black hollyhock extract; ME‐BE, microencapsulated borage extract; ME‐BHE, microencapsulated black hollyhock extract.

Color is a noticeable sensory parameter of food products acceptability for the consumer. Figure 4 illustrates the color variations observed in noodles with different additives. The color indexes of instant fried noodles, including L* (lightness), a* (redness–greenness), b* (yellowness–blueness), and Δ*E* (total color difference), are presented in Table 4 Clearly, the incorporation of free and encapsulated BE and BHE had a significant effect on the color indexes of fried noodles (*p* < .05). As expected, by adding these extracts, the color of the noodles became darker, the redness decreased, and the yellowness increased (*p* < .05). The effect of free extracts on changing the color of noodles was greater than that of microencapsulated extracts. The Δ*E* values of the noodles enriched with free extracts were also significantly higher than those of encapsulated extracts. The presence of natural pigments, especially anthocyanins and carotenoids in borage and black hollyhock, is the reason for these color changes in enriched noodles. The L*, a*, b*, and Δ*E* values of fried noodle samples were in the range of 59.17 to 72.34, −1.02 to −6.17, 1.86 to 4.38, and 4.77 to 14.18, respectively. The darker color of the noodles due to adding persimmon juice was also reported by Han et al. (2012). Similarly, in Khare et al. (2014), adding mint oil and eugenol led to a darker color of the noodles compared to the control. In another study, adding borage leaf extract significantly decreased the L* of pasta samples and caused a decrease in a* and a slight increase in b* of cooked samples. These color changes were acceptable from the point of view of consumers (Miceli et al., 2015).

**TABLE 4:** Color properties of instant fried noodle samples.

Samples	L*	a*	b*	Δ*E*
Control	72.34 ± 1.01^a^	−1.02 ± 0.25^a^	1.86 ± 0.22^c^	‐
BE	60.10 ± 1.18^c^	−3.51 ± 0.17^c^	4.38 ± 0.27^a^	12.74 ± 0.54^b^
BHE	59.17 ± 0.89^c^	−6.17 ± 0.12^d^	2.95 ± 0.21^b^	14.18 ± 0.66^a^
ME‐BE	67.65 ± 0.97^b^	−1.39 ± 0.19^a^	1.98 ± 0.16^c^	4.77 ± 0.42^b^
ME‐BHE	66.73 ± 1.05^b^	−2.26 ± 0.23^b^	2.57 ± 0.19^b^	5.79 ± 0.50^c^

*Note*: Values represent the mean (*n* = 3) ± SD. Different letters in each column show a significant difference (*p* < .05).

Abbreviations: BE, borage extract; BHE, black hollyhock extract; ME‐BE, microencapsulated borage extract; ME‐BHE, microencapsulated black hollyhock extract.

### 3.6 TPA of instant fried noodles

Texture is an important parameter of instant noodles that affects the acceptability of the product (Jin et al., 2021). The results of the TPA of the instant fried noodles (Table 5) showed that the incorporation of free and microencapsulated BE and BHE into the noodle formulation did not have any significant impact on the texture profile of the produced noodles and only led to a slight decrease in the amount of adhesiveness. This slight reduction in adhesiveness due to the addition of extracts was also reported by Marinelli et al. (2015). It is attributed to the creation of a strong link between the phenolic compounds in the extracts and the gluten network, which traps the starch granules in this strengthened structure and reduces the release of amylose from the product structure. The hardness, adhesiveness, springiness, gumminess, and chewiness values of fried noodles were in the 1.25–1.41 N, 0.03–0.07, 4.00–4.22 cm, 0.63–0.69 N, and 2.75–3.09 N/cm ranges, respectively. Pasqualone et al. (2017) also found that the addition of artichoke canning by‐product extract did not have any significant impact on the pasta texture. In Kazemi et al. (2017), adding different levels of pomegranate peel extract to the yellow alkaline noodle formulation only resulted in a small increase in the hardness of the noodles; however, the texture changes were not significant.

**TABLE 5:** Textural properties of instant fried noodle samples.

Samples	Hardness (N)	Adhesiveness	Springiness (cm)	Gumminess (N)	Chewiness (N/cm)
Control	1.27 ± 0.09^a^	0.07 ± 0.03^a^	4.02 ± 0.12^a^	0.69 ± 0.07^a^	2.75 ± 0.34^a^
BE	1.36 ± 0.18^a^	0.03 ± 0.01^a^	4.00 ± 0.17^a^	0.68 ± 0.11^a^	3.00 ± 0.14^a^
BHE	1.41 ± 0.16^a^	0.03 ± 0.02^a^	4.22 ± 0.13^a^	0.63 ± 0.07^a^	3.09 ± 0.21^a^
ME‐BE	1.25 ± 0.14^a^	0.03 ± 0.03^a^	4.15 ± 0.07^a^	0.65 ± 0.15^a^	2.88 ± 0.25^a^
ME‐BHE	1.29 ± 0.11^a^	0.04 ± 0.02^a^	4.07 ± 0.10^a^	0.69 ± 0.05^a^	2.93 ± 0.14^a^

*Note*: Values represent the mean (*n* = 3) ± SD. Different letters in each column show a significant difference (*p* < .05).

Abbreviations: BE, borage extract; BHE, black hollyhock extract; ME‐BE, microencapsulated borage extract; ME‐BHE, microencapsulated black hollyhock extract.

### 3.7 Oxidation of fried noodles during storage period

#### 3.7.1 AV of fried noodles

**FIGURE 5:**
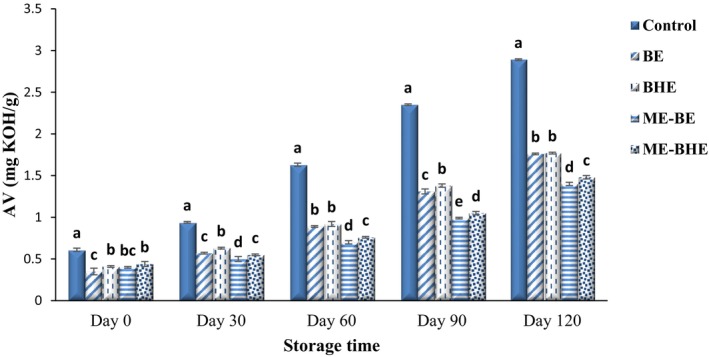
Changes in AV (mg KOH/g) of instant fried noodle samples during the storage period. Bars represent the mean (*n* = 3) ± SD. Different letters on the bars show a significant difference (*p* < .05). BE, borage extract; BHE, black hollyhock extract; ME‐BE, microencapsulated borage extract; ME‐BHE, microencapsulated black hollyhock extract.

The effects of free and microencapsulated BE and BHE on the AV of fried noodles during the 120‐day storage period at room temperature are shown in Figure 5. During the storage period, a significant increase in the AV of the noodle samples was observed (*p* < .05), so that the AV values of samples were in the range of 0.35–0.61 mg KOH/g on the first day of the experiments and reached 1.40–2.89 mg KOH/g on the 120th day of storage. The increase in the AV of the noodles during the storage period is related to the hydrolysis of triglycerides by the lipase enzyme. Since these free fatty acids participate in auto‐oxidation or enzymatic oxidation and cause the degradation of the quality of the food product, decreasing their amounts is desirable (Jia et al., 2021). As expected, without antioxidants in the control sample, the highest increase in AV was observed in this sample, and the addition of free and microencapsulated BE and BHE resulted in a significant decline in the intensity of triglyceride hydrolysis in the noodle samples (*p* < .05). At the beginning, there was not any significant difference in the AV of the noodles containing free and microencapsulated extracts; however, on the last day of storage, the samples containing microencapsulated extracts had the lowest AV. In Deora et al. (2016), adding rosemary extract significantly reduced the production of free fatty acids during the frying process of noodles compared to the control samples. Malekhossini et al. (2021) found that the use of encapsulated beta‐carotene in oil samples could reduce the intensity of triglyceride hydrolysis in the samples and result in a significant decline in the AV of the enriched oils. Hosseinialhashemi et al. (2021) also obtained similar results regarding the effects of *Pistacia khinjuk* extract nanoemulsion on the oil's stability.

#### 3.7.2 POV of fried noodles

**FIGURE 6:**
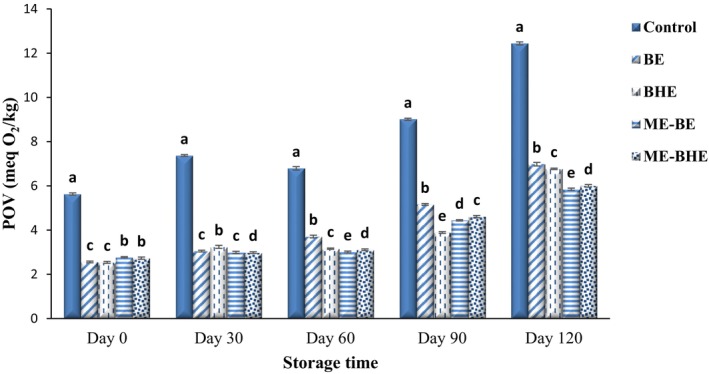
Changes in peroxide value (POV) values (meq O_2_/kg) of instant fried noodle samples during the storage period. Bars represent the mean (*n* = 3) ± SD. Different letters on the bars show a significant difference (*p* < .05). BE, borage extract; BHE, black hollyhock extract; ME‐BE, microencapsulated borage extract; ME‐BHE, microencapsulated black hollyhock extract.

The peroxide index is a qualitative index of the oxidation of fats or oils in primary products. Hydroperoxides are continuously produced by this process, and due to their instability, they decompose and produce secondary products of oxidation (Boroujeni & Hojjatoleslamy, 2018). The effect of free and microencapsulated BE and BHE on the POV of fried noodles during a 120‐day storage period at room temperature is shown in Figure 6. A significant increase in POV of the noodle samples was seen (*p* < .05) during the storage period. The POV of samples was in the range of 2.54–5.63 meq O_2_/kg on the first day of the experiments and reached 5.85–12.44 meq O_2_/kg on the 120th day of storage. By adding free and microencapsulated BE and BHE to the noodle formulation, a significant decline was observed in the POV of the samples in comparison with the control sample (*p* < .05), which was related to the remarkable antioxidant activity of these extracts. At the end of the storage period, the antioxidant activity of microencapsulated extracts was higher than that of free extracts, which was related to the protective role of the encapsulation process on bioactive compounds. In general, herbal extracts contain bioactive compounds that increase the stability of radicals by donating hydrogen or electrons to unstable radicals, thereby delaying lipid oxidation. Bioactive compounds such as polyphenols also have the property of neutralizing ROS (Liu & Yao, 2007). In line with these results, Song and Yoo (2017) observed a decrease in the production rate of hydroperoxides due to the addition of green tea extract to the instant noodle formulation. Jafari et al. (2022) also reported the significant effect of micro‐ and nano‐capsules of rosemary in reducing the POV of oil during the storage period and reported that the effects of encapsulated extracts were higher than the free extract. These results are consistent with the findings by Yazdan‐Bakhsh et al. (2021) and Kazemi et al. (2017).

#### 3.7.3 TBA of fried noodles

**FIGURE 7:**
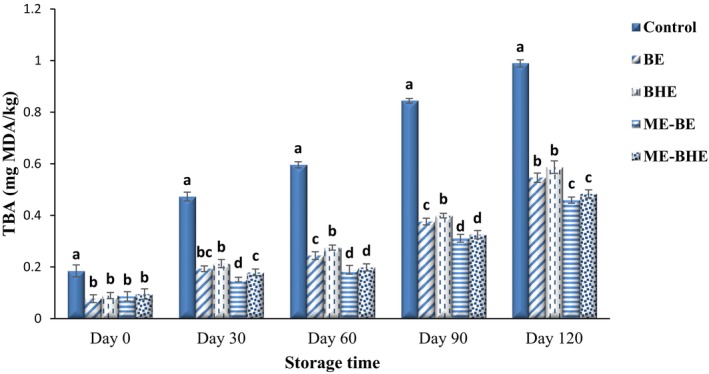
Changes in thiobarbituric acid (TBA) values (mg MDA/kg) of instant fried noodle samples during the storage period. Bars represent the mean (*n* = 3) ± SD. Different letters on the bars show a significant difference (*p* < .05). BE, borage extract; BHE, black hollyhock extract; ME‐BE, microencapsulated borage extract; ME‐BHE, microencapsulated black hollyhock extract.

As stated before, hydroperoxides, which are the primary products of oxidation, are unstable and decompose into secondary products (such as free fatty acids, ketones, aldehydes, and alcohol). TBA is one of the most widely used oxidation indexes to determine secondary products of lipid oxidation in food products (Silva Faria et al., 2020). The effects of free and microencapsulated BE and BHE on the TBA of fried noodles during the 120‐day storage period at room temperature are shown in Figure 7. Since malondialdehyde is formed through the decomposition of hydroperoxides, as a result, gradually and with the further decomposition of hydroperoxides and conversion into secondary products, TBA increases significantly (*p* < .05). On the first day of storage, the TBA of the noodle samples was in the range of 0.077–0.185 mg MDA/kg, and on the last day of storage, it increased to 0.459–0.989 mg MDA/kg. As expected, on different storage days, the highest TBA was seen in the control sample, and the use of free and microencapsulated BE and BHE in the noodle formulation significantly decreased the TBA (*p* < .05). Phenolic compounds demonstrate remarkable antioxidant activity due to their capability to neutralize free radicals, chelate metal ions, and inhibit oxidative enzymes (Nieto, 2020). Since the encapsulation process increases the stability of the bioactive compounds and their controlled release by creating a protective coating around these compounds (Sharma et al., 2019), the samples containing microencapsulated extracts maintained their antioxidant activity for a longer period of time. This caused a higher reduction in lipid oxidation in fried noodles. In line with these results, Khare et al. (2014) also reported the significant effect of eugenol on reducing the amount of malondialdehyde in noodle samples. The higher antioxidant effect of encapsulated extracts compared to free extracts was also observed by Jafari et al. (2022). Similarly, Yazdan‐Bakhsh et al. (2021) found the encapsulated *H. lasiopetalum* extract with whey protein isolate to have a higher antioxidant activity than the free extract, especially in the last days of storage.

#### 3.7.4 AI of fried noodles

**FIGURE 8:**
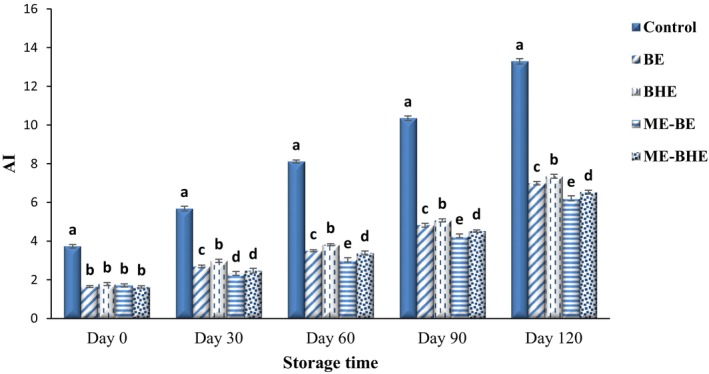
Changes in anisidine index (AI) values of instant fried noodle samples during the storage period. Bars represent the mean (*n* = 3) ± SD. Different letters on the bars show a significant difference (*p* < .05). BE, borage extract; BHE, black hollyhock extract; ME‐BE, microencapsulated borage extract; ME‐BHE, microencapsulated black hollyhock extract.

AI is another oxidative index to measure the secondary products of lipid oxidation. The effects of free and microencapsulated BE and BHE on the AI of fried noodles during the 120‐day storage period at room temperature are shown in Figure 8. During the storage period, a significant increase in AI of the noodle samples was observed (*p* < .05), so that the AI values of samples were in the range of 1.65–3.73 on the first day and reached 6.21–13.29 on the 120th day of storage. Throughout the storage, the highest amount of AI was observed in the samples without antioxidants (control). The BE and BHE indicated remarkable antioxidant activity in fried noodles, and the activity of their microencapsulated form was higher than the free form. These results were in agreement with Hosseinialhashemi et al. (2021) and Delfanian et al. (2018) in terms of the reduction of oxidation products of oils due to the incorporation of encapsulated extracts. A reduction in the AI of oil samples due to the use of different herbal extracts was also observed by Hassan, El‐Sayed Hassan et al. (2022).

#### 3.7.5 CDs of fried noodles

**FIGURE 9:**
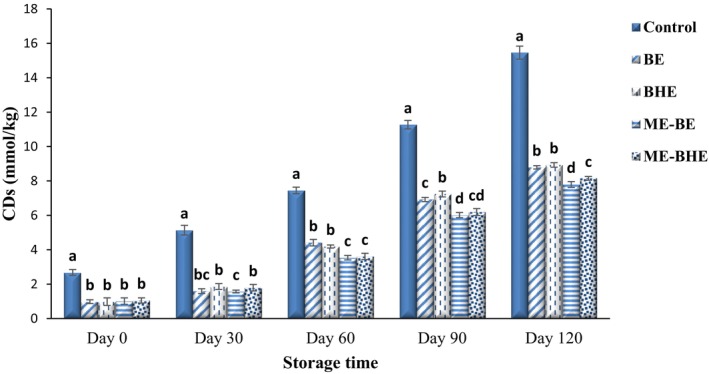
Changes in conjugated diene (CD) values (mmol/kg) of instant fried noodle samples during the storage period. Bars represent the mean (*n* = 3) ± SD. Different letters on the bars show a significant difference (*p* < .05). BE, borage extract; BHE, black hollyhock extract; ME‐BE, microencapsulated borage extract; ME‐BHE, microencapsulated black hollyhock extract.

During lipid oxidation, as a result of removing a hydrogen atom from polyunsaturated fatty acids by ROS, a double bond next to the oxygen‐free carbon was transferred to another double bond. This displacement formed CDs, which led to the stabilization of the molecule. The CDs test was used to study the rate of lipid oxidation in the early stages of oxidation (Abeyrathne et al., 2021). The effect of free and microencapsulated BE and BHE on the CDs of fried noodles during the 120‐day storage period at room temperature is shown in Figure 9. During the storage period and as a result of the development of lipid oxidation, a significant increase happened in CDs values of fried noodles (*p* < .05), and the highest rate of increase was seen in the control sample. The CD value of the control samples was 2.68 mmol/kg at the beginning and increased to 15.46 mmol/kg on the last day. In the noodles enriched with BE and BHE, the CD values were in the range of 0.98–1.05 mmol/kg on the first day and reached 7.79–8.93 mmol/kg on the last day of storage. Neves et al. (2020) reported the effect of alpha‐tocopherol encapsulated with whey protein on increasing the oxidative stability of oil and reducing the CDs values in oil samples. Malekhossini et al. (2021) also showed a decrease in the CDs in oil samples due to the incorporation of beta‐carotene microcapsules.

### 3.8 Sensory evaluation of fried noodles

**FIGURE 10:**
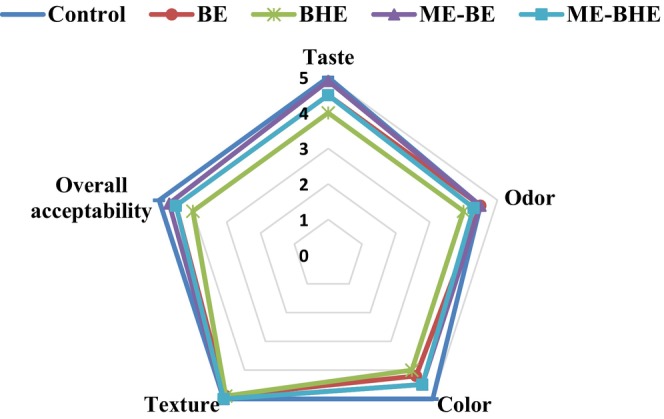
Sensory profile of the instant fried noodle samples.

The results of the sensory evaluation of instant fried noodles are presented in Table 6 and Figure 10. Adding free and microencapsulated BE and BHE to the noodle formulation significantly decreased the taste, color, and overall acceptability scores of noodle samples. However, it did not have a significant effect on the texture score of the noodles. In terms of odor, only the sample containing free BHE had a significant difference from the other samples and obtained a low score. In general, despite the decrease in the sensory scores of the noodle samples due to the addition of free and microencapsulated BE and BHE, all the samples examined in this work scored high and were acceptable from the point of view of the panelists. Miceli et al. (2015) found that pasta samples containing borage leaf extracts had a high level of sensory acceptance. Khare et al. (2014) observed that adding eugenol improved the sensory acceptance of noodles, while peppermint oil decreased the overall acceptance score of samples. High sensory acceptance of pasta enriched with polyphenol grape extract was also reported by Marinelli et al. (2015). Kazemi et al. (2017) showed that adding pomegranate peel extract did not have any negative impact on the sensory acceptance of cooked noodles.

**TABLE 6:** Sensory scores of instant fried noodle samples.

Samples	Taste	Odor	Color	Texture	Overall acceptability
Control	5.00 ± 0.00^a^	4.50 ± 0.26^a^	5.00 ± 0.00^a^	5.00 ± 0.00^a^	5.00 ± 0.00^a^
BE	4.50 ± 0.36^b^	4.50 ± 0.36^a^	4.20 ± 0.24^bc^	4.90 ± 0.16^a^	4.50 ± 0.36^b^
BHE	4.00 ± 0.00^c^	4.00 ± 0.00^b^	4.00 ± 0.00^c^	4.90 ± 0.16^a^	4.00 ± 0.00^c^
ME‐BE	4.90 ± 0.16^ab^	4.50 ± 0.26^a^	4.50 ± 0.36^b^	5.00 ± 0.00^a^	4.70 ± 0.16^b^
ME‐BHE	4.50 ± 0.36^b^	4.30 ± 0.26^a^	4.50 ± 0.36^b^	5.00 ± 0.00^a^	4.50 ± 0.36^b^

*Note*: Values represent the mean (*n* = 3) ± SD. Different letters in each column show a significant difference (*p* < .05).

Abbreviations: BE, borage extract; BHE, black hollyhock extract; ME‐BE, microencapsulated borage extract; ME‐BHE, microencapsulated black hollyhock extract.

## 4 CONCLUSION

The results of this study showed that adding free and microencapsulated BE and BHE to the noodle formulation led to a decrease in cooking loss and pH of the samples and darkened the color of the instant fried noodles. The negative impact of microencapsulated extracts on the color indexes and sensory characteristics of the noodles was significantly reduced compared to the free extracts. The fried noodles enriched with free and microencapsulated BE and BHE had a higher oxidative stability than the noodles without extract and also obtained high sensory scores. The microencapsulation process with whey protein isolates and maltodextrin improved the stability of the bioactive compounds in fried noodle samples compared to the free extracts, and its effects were more evident in the longer terms. Therefore, the oxidative stability and cooking loss of instant fried noodles can be improved through enriching the noodle formulation with BE and BHE, especially in microencapsulated form. As the noodle samples containing encapsulated BE and BHE exhibited the lowest oxidative indexes, they were selected as the most favorable treatments in this study. Consequently, employing microencapsulated BE and BHE is recommended to enhance the oxidative stability of fried food products throughout the processing and storage phases.

## AUTHOR CONTRIBUTIONS

**Mahshid Zamankhani:** Conceptualization (equal); funding acquisition (equal); investigation (equal); methodology (equal); visualization (equal); writing – original draft (equal); writing – review and editing (equal). **Sohrab Moeini:** Project administration (equal); supervision (equal); validation (equal); writing – review and editing (equal). **Peyman Mahasti Shotorbani:** Conceptualization (equal); methodology (equal); project administration (equal); supervision (equal). **Hossein Mirsaeedghazi:** supervision (equal); validation (equal); **Afshin Jafarpour:** supervision (equal); validation (equal).

## CONFLICT OF INTEREST STATEMENT

The authors declare that the research was carried out in the absence of any commercial or financial relationships that could be construed as a conflict of interest.

## Data Availability

The data supporting the findings of this study are available from the corresponding author upon reasonable request.
